# Potential of Drought Tolerant Rhizobacteria Amended with Biochar on Growth Promotion in Wheat

**DOI:** 10.3390/plants13091183

**Published:** 2024-04-24

**Authors:** Sidra Noureen, Atia Iqbal, Hafiz Abdul Muqeet

**Affiliations:** 1 Department of Microbiology and Molecular Genetics, The Women University, Multan 66000, Pakistan; sidranoureen911@gmail.com; 2 Department of Electrical Engineering and Technology, Punjab Tianjin University of Technology, Lahore 53720, Pakistan

**Keywords:** biochar, bio-formulation, carrier material, drought stress, PGPR, wheat

## Abstract

Drought stress is the prime obstacle for worldwide agricultural production and necessitates innovative strategies for enhancing crop resilience. This study explores the efficacy of plant growth-promoting rhizobacteria (PGPR) and biochar (BC) as sustainable amendments for mitigating the effects of drought on wheat growth. Multiple experiments were carried out on isolated strains to assess their drought tolerance potential and multiple plant growth-promoting attributes. Experiments in the laboratory and natural environment were conducted to assess the impact of plant growth-promoting rhizobacteria, biochar, and their synergistic application on various growth parameters of wheat. The results revealed that the drought-tolerant PGPR strains (*Bacillus subtilis* and *Bacillus tequilensis*), alongside biochar (rice husk), alleviated the phytotoxic impact of drought by increasing the root length from 17.0% to 70.0% and shoot length from 30.0% to 82.0% as compared to un-inoculated stressed controls. The total chlorophyll and carotenoid contents of the plants were substantially increased to 477% and 423%, respectively, when biochar and PGPR were applied synergistically. Significant enhancements in membrane stability index, relative water content, proline, and sugar level were achieved by combining biochar and bacterial strains, resulting in increases of 19.5%, 37.9%, 219%, and 300%, respectively. The yield of wheat in terms of plant height, spike length, number of spikelets per spike, and number of grains per spike was enhanced from 26.7% to 44.6%, 23.5% to 62.7%, 91.5% to 154%, and 137% to 182%, respectively. It was concluded that the biochar-based application of PGPR induced drought tolerance in wheat under water deficit conditions, ultimately improving the production and yield of wheat.

## 1. Introduction

Drought stress has a rigorous effect on plant growth and productivity and significantly impedes crop throughput. Overall, 39% of the world’s residents live in regions with up to 45% of their agricultural land suffering from regular or persistent drought. The lack of precipitation, excessive heat, poor reservoir capacity, and global warming have all contributed to this issue for decades [1]. Drought-affected agricultural regions may lose up to 50% or more of their yield [2]. Agriculture is the main industry in Pakistan that is impacted by the growing scarcity of fresh water, as the country’s economy is centered on this sector [3]. Globally, Pakistan falls sixth in the list of most drought-susceptible countries, with Sindh alone accounting for almost 2.5 million people who are impacted [4]. By 2025, the scenario could be more concerning because the aforementioned factors affect around 15 million hectares of agricultural land in Pakistan, resulting in decreased output [5].

Reactive oxygen species (ROS) can build up in wheat (*Triticum aestivum*) when it is under drought stress [6]. Overexposure to ROS results in oxidative stress, which, in turn, leads to the manufacture of antioxidants to relieve water stress [7]. Conditions that restrict water are also linked to negative effects on the photosynthetic qualities of crops. Additionally, the cycling of nutrients in the soil and cop accessibility are negatively impacted by water stress [8].

Drought has a severe impact on the growth and yield of wheat, leading to crop failure [9]. Genetic engineering is used to develop drought-tolerant varieties of wheat, which involves multiple genes [10]. Due to the involvement of multiple genes in this process, it becomes difficult to develop the desired phenotype [11]. Typically, farmers and the public do not prefer transgenic varieties due to their ecological danger and processing times. The use of carrier-based bacterial formulations is suggested as an alternative to transgenic crops, as it is a cost-effective and eco-friendly strategy to alleviate drought stress. PGPR have extensive applications in agriculture as an alternative to chemical and nutrient fertilizers [12]. The use of beneficial bacteria assuages drought stress in wheat by increasing root length, which, in turn, enhances the uptake of water [13].

Bacteria produce auxin, gibberellins, siderophores [14], and biosurfactants to escape from the detrimental effects of drought. Bacteria also produce osmolytes to reduce the harmful effects of abiotic stresses [15]. EPS-producing bacteria develop biofilms and protect bacteria from dehydration during drought stress [16].

Using bacterial inoculum directly in soil has many ecological limitations, including the short shelf lives of these inoculums and environmental leakage [17]. Thus, there is a need for efficient carrier materials that can support bacterial survival and development. A carrier material enhances the shelf life of an inoculum. Because they are simple to handle and can be preserved for a long time, carrier-based bacterial inoculants are effectively used in different crops [18].

Biochar is prepared from the pyrolysis of organic material which is rich in carbon content [19] and porous. Biochar has been extensively utilized as a soil amendment under water stress conditions to enhance the yield of wheat [20]. By preserving membrane stability, enhancing water absorption, preserving nutritional homeostasis, and through increased antioxidant activities, biochar significantly increases tolerance to drought stress in wheat [21]. Furthermore, the enhancement of soil properties through BC-mediated means also significantly boosts the activity of photosynthetic processes and chlorophyll synthesis. Additionally, it preserves hormonal balance and osmolytes, all of which enhance resistance to osmotic stress.

It has a porous surface that improves the water retention of the soil, microbial development, and crop nutrition and provides relief from abiotic stresses [22].

Upon an in-depth review of the previously listed facts, the application of drought-tolerant PGPR and rice husk biochar in different soils has been frequently investigated to combat drought effects in pot experiments under laboratory and natural conditions. However, evaluations of their synergistic effect in drought stress mitigation and the promotion of wheat growth under controlled laboratory and natural environment-based conditions remain limited. Therefore, the objective of this study was to observe the efficiency of the combined application of drought-tolerant PGPR and rice husk biochar in granting resistance and growth promotion in wheat crops against drought impacts. This study also evaluates the shelf life of PGPR in biochar. We hypothesized that seed coating with drought-tolerant PGPR, along with biochar, would be a more efficient technique for mitigating adverse drought effects on wheat growth and yield traits.

## 2. Results

### 2.1. Isolation of Rhizobacteria

A total of 42 bacterial strains were isolated from various rhizospheric samples. Bacterial isolates were selected on the basis of their distinct morphological features. All isolated bacterial colonies were smooth and shiny, ranging from small in size to medium and large.

### 2.2. Isolation of Drought-Tolerant Bacterial Isolates

**Figure 1:**
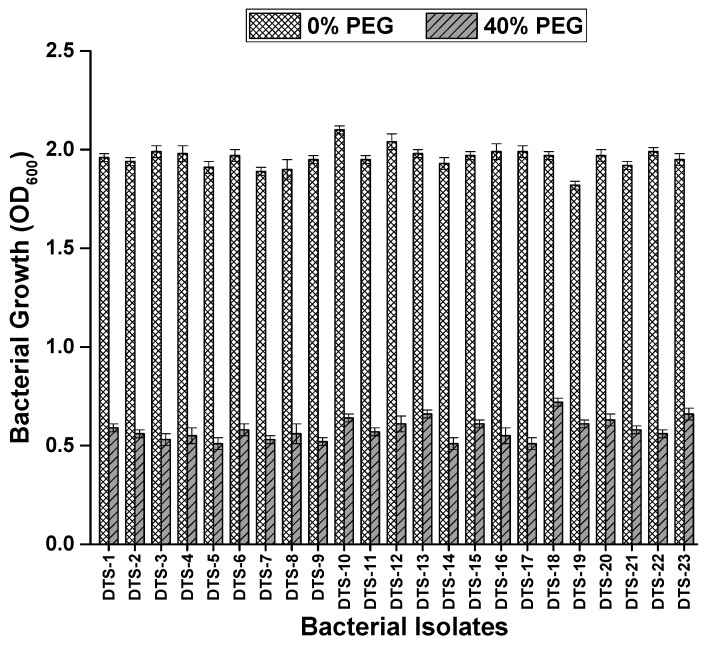
Growth of bacterial strains in terms of optical density under normal conditions and drought stress (0% and 40% polyethylene glycol). Bars represent standard error.

Drought stress was applied to the bacteria strains to check their drought tolerance potential. Among the 42 bacterial isolates, 11 isolates appeared sensitive to drought stress. Eight isolates showed moderate tolerance to drought stress. Twenty-three (23) isolates were able to grow at 40% PEG (Figure 1). These 23 isolates were classed as drought-tolerant strains (DTS) and were utilized in further experiments. The majority of these colonies were glossy and smooth. The sizes of the colonies varied between small, medium, and large. About 21.9% of the bacterial cells were vibrio and cocci, and 72.0% of the cells were rod-shaped. Bacterial strains stained with Gramm stain showed that 56.5% were Gram-positive and 43.5% were Gram-negative.

### 2.3. PGP Traits of Drought-Tolerant Rhizobacteria

A series of selection rounds were conducted on the isolates to identify the best PGPR in terms of their potential as bio-fertilizers and active components. Various PGP properties were assessed for this aim, such as phosphate solubilization, EPS production, ammonia production, HCN production, the secretion of plant hormones, and siderophore production.

**Figure 2:**
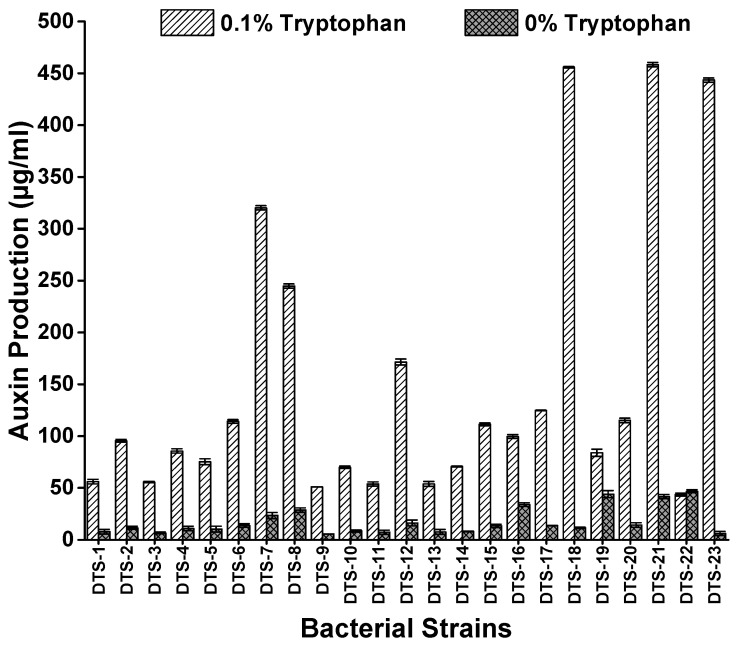
Auxin production by drought-tolerant rhizobacteria with and without amendment of L-tryptophan. Bars represent standard error.

All strains showed different potentials of auxin production ranging from 4.61 μg/mL (DTS-22) to 45.8 μg/mL (DTS-23) without tryptophan and 43.7 μg/mL (DTS-22) to 458 μg/mL (DTS-21) when culture media was amended with 0.1% tryptophan. The highest auxin-producing strains were DTS-21, DTS-18, and DTS-23, which produced 458 μg/mL, 456 μg/mL, and 443 μg/mL auxin, respectively (Figure 2, Table 1).

**Figure 3:**
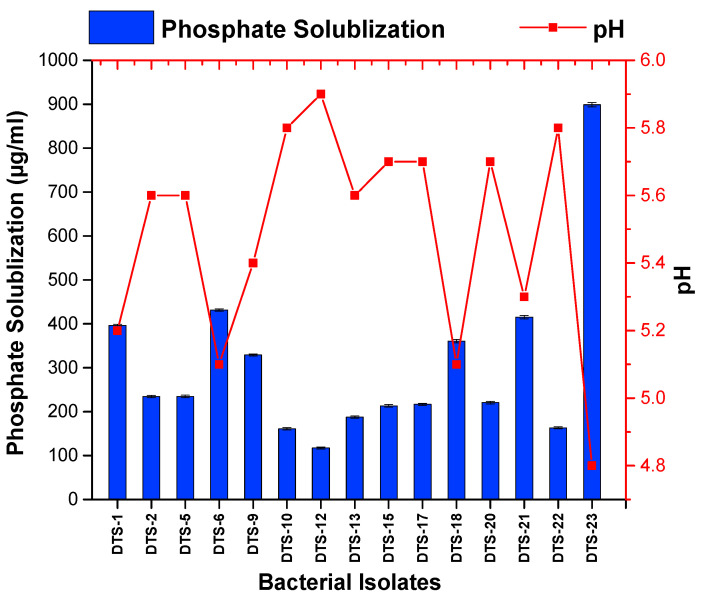
Phosphate solubilization by drought-tolerant rhizobacteria. Bars represent standard error.

Among the 23 drought-tolerant rhizobacteria, 15 bacteria revealed the capacity to solubilize the inorganic phosphate in media. However, 8 bacteria appeared as negative in the phosphate solubilization test. The range of the phosphate solubilization index (PSI) fell between 2.1 and 4.75. The highest PSI value was shown by strain DTS-23 (4.75), followed by DTS-21 (4.75) and DTS-18 (3.8). All other strains showed less than 3 PSI. The quantitative phosphate solubilization ability ranged from 117 μg/mL to 899 μg/mL. The pH of the media also changed with phosphate solubilization and ranged from 4.8 to 5.1 (Figure 3, Table 1).

**Figure 4:**
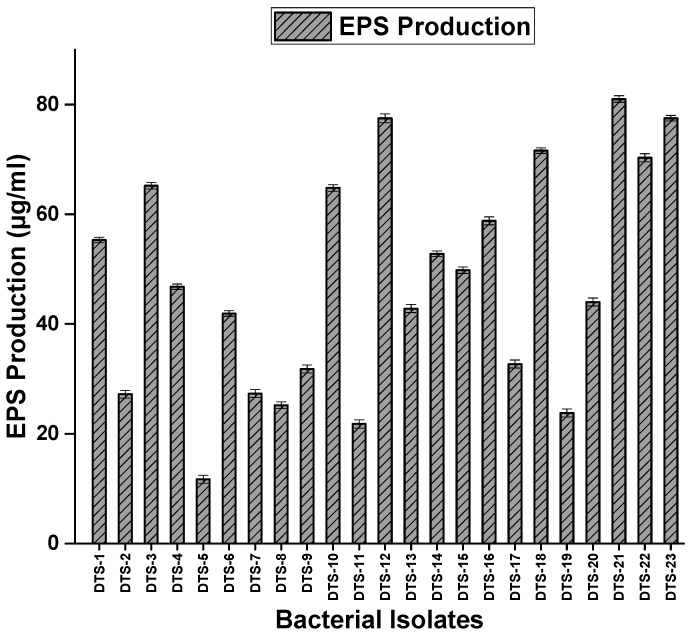
Exopolysaccharide production by drought-tolerant rhizobacteria. Bars represent standard error.

All drought-tolerant strains showed the production of exopolysaccharide in the form of mucoid colonies. Different strains showed different potentials for EPS production. Strains DTS-22, DTS-16, and DTS-19 produced the highest amount of EPS (83.8 μg/mL, 87.0 μg/mL, and 81.0 μg/mL, respectively). DTS-5 showed the lowest production of EPS (11.8 μg/mL) among all strains (Figure 4, Table 1). Only 52% of isolates were able to grow on nitrogen-free media by fixing nitrogen. DTS-18, DTS-21, and DTS-23 were the best nitrogen-fixing strains. Fifty-six percent (56%) of the isolates were able to produce HCN, and DTS-21 and DTS-22 were the best HCN producers. Fifty-two percent (52%) of the bacteria produced ammonia.

**Figure 5:**
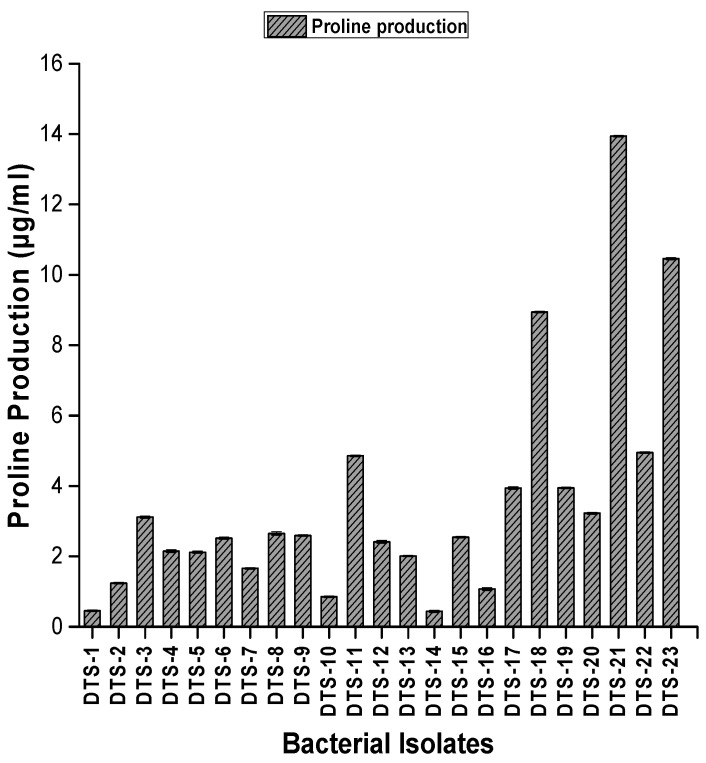
Proline production by drought-tolerant rhizobacteria. Bars represent standard error.

**Figure 6:**
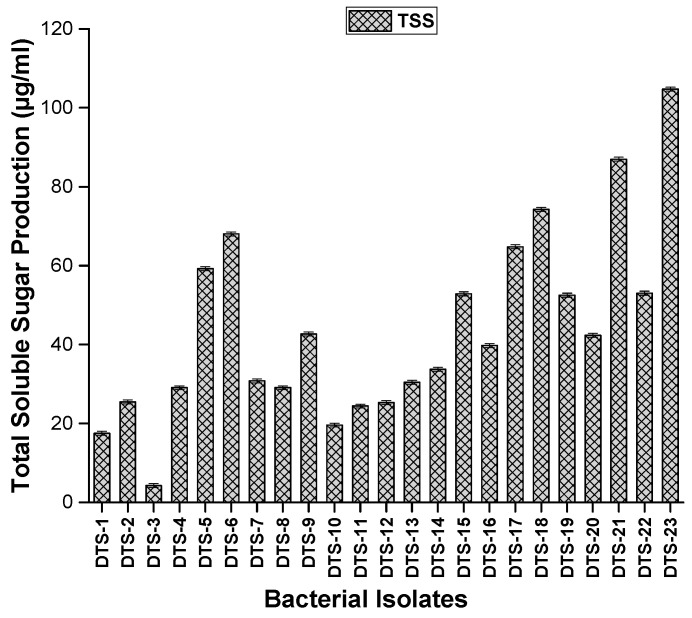
Total soluble sugar (TSS) production by drought-tolerant rhizobacteria. Bars represent standard error.

Traits associated with oxidative stress, such as the production of proline and total soluble sugar, were evaluated by growing bacterial isolates in LB broth. Regarding total soluble sugar production, all drought-tolerant isolates produced total soluble sugar ranging from 4 μg/mL to 105 μg/mL. DTS-23 and DTS-21 produced the highest amount of soluble sugar, i.e., 105 μg/mL and 86.1 μg/mL, respectively (Figure 5). All strains were positive for proline production. Proline production ranged from 0.44 μg/mL to 13.9 μg/mL. DTS-19 and DTS21 produced the highest amounts of proline: 13.9 μg/mL and 10.4 μg/mL, respectively (Figure 6).

The extracellular enzyme production abilities, pertaining to enzymes such as amylase, lipase, pectinase, chitinase, and protease, of the PGPR were also evaluated. Regarding protease and chitinase production, 34% of isolates were positive for protease and chitinase production. Pectinase and lipase production was shown only by 52% of strains. Forty-eight percent (48%) of the isolates were able to produce amylase enzyme.

The three most efficient strains (DTS-19, DTS-21, and DTS-23), which were positive for most of the PGP traits, were used for the assessment of their potential for promoting the growth of wheat under laboratory and natural conditions.

### 2.4. Morphological, Biochemical, and Molecular Characterization of Bacterial Strains

**Figure 7:**
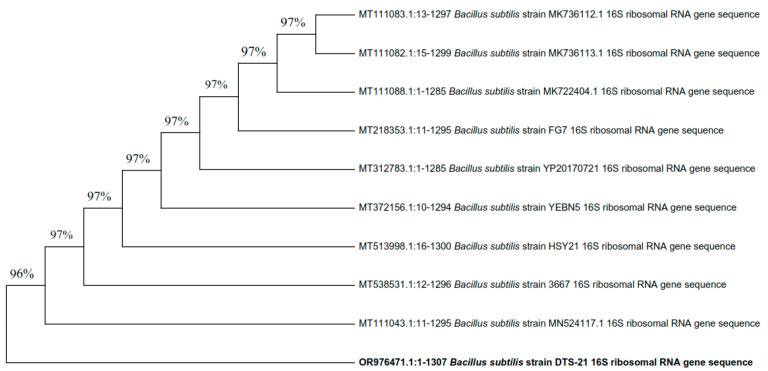
Phylogenetic tree of drought-tolerant rhizobacterial strain DTS-21 and known plant growth-promoting rhizobacteria.

**Figure 8:**
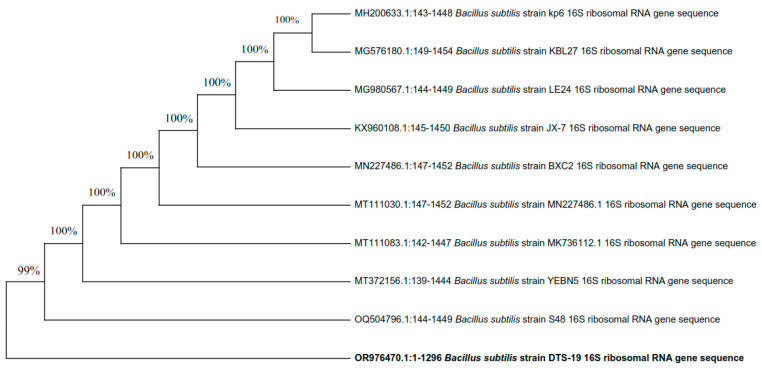
Phylogenetic tree of drought-tolerant rhizobacterial strain DTS-19 and known plant growth-promoting rhizobacteria.

**Figure 9:**
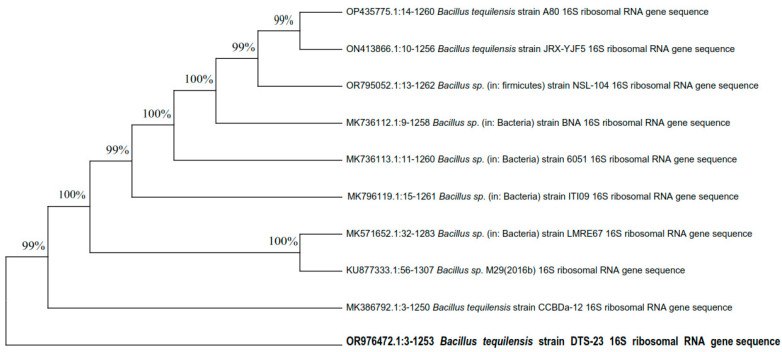
Phylogenetic tree of drought-tolerant rhizobacterial Strain DTS-23 and known plant growth-promoting rhizobacteria.

The three most efficient strains (DTS-19, DTS-21, and DTS-23) which were positive for most of the PGP traits appeared as Gram-positive rods. All three bacterial isolates can synthesize the enzymes catalase and amylase and use citrate. Based on their visual and biochemical traits, DTS-19, DTS-21, and DTS-23 were identified as *Bacillus* sp. Additionally, 16S rRNA gene sequencing was used for the confirmation of the bacterial species. Isolates DTS-19, DTS-21, and DTS-23 were identified as *B. subtilis*, *B. subtilis,* and *B. tequilensis* (GenBank Accession Numbers: OR976470, OR976571, and OR976472) based on their phylogenetic trees and nucleotide homology produced by the neighbor-joining method (Figure 7, Figure 8 and Figure 9).

### 2.5. Characterization of Biochar and Survival Percentage of PGPR in Biochar

Our physicochemical analysis of biochar showed 15.8% ash content, pH 7.8, 2.19 EC (dS-1), and moisture content. The total nitrogen content was 1.98%. Total P and K were measured at 2.01 g/kg and 2.07 g/kg, respectively (Table 2).

During the storage test at 25 °C for 6 months, the bacterial concentrations in biochar remained relatively consistent during the initial 3 months, with a bacterial population of 21.3 × 10^8^, 22.3 × 10^8^, and 28.3 × 10^8^ CFU/mg for strains DTS-19, DTS-21, and DTS-23, respectively (Table 3). However, beyond the 3-month mark, there was a gradual decrease in CFU counts, culminating in population levels of 2.3 × 10^8^, 3.6 × 10^8^, and 12.3 × 10^8^ CFU/mg for strains TDTS-19, DTS-21, and DTS-23, respectively, by the 6th month.

### 2.6. Vegetative Attributes of the Wheat Plant

**Figure 10:**
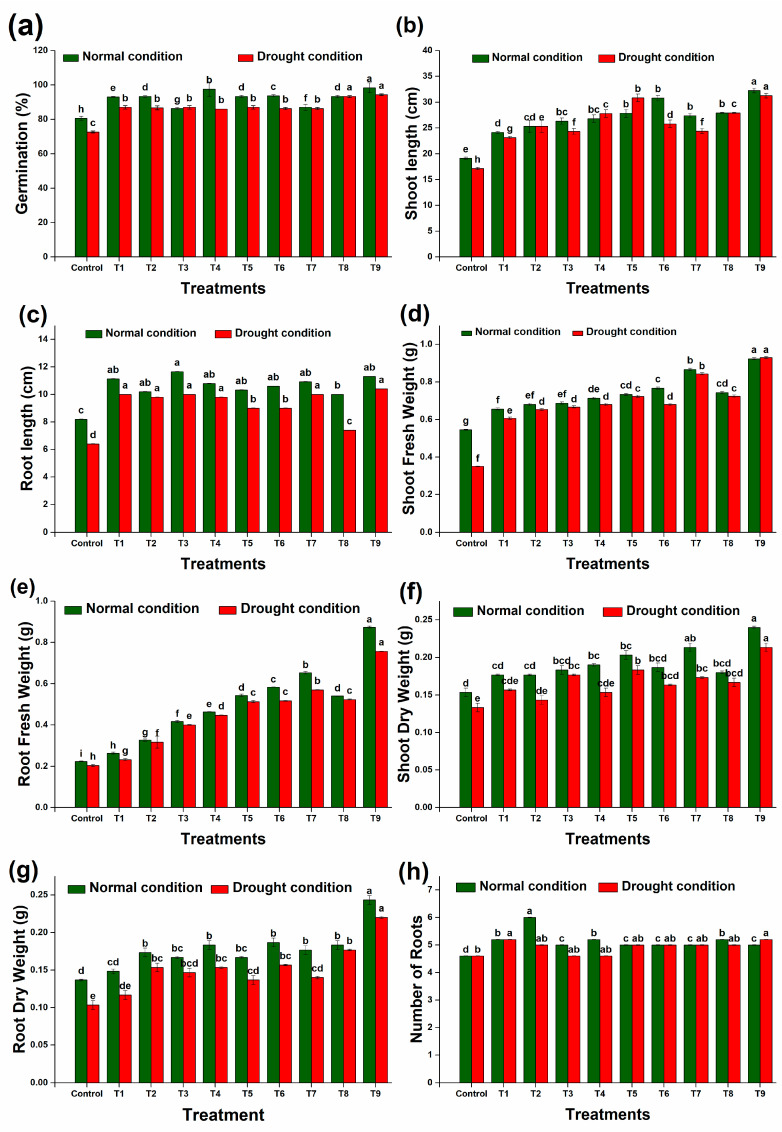
Effect of different treatments on physical parameters of wheat plants: (**a**) germination, (**b**) shoot length, (**c**) root length, (**d**) shoot fresh weight, (**e**) root fresh weight, (**f**) shoot dry weight, (**g**) root dry weight, and (**h**) number of roots. Data are presented as means of 3 replicates. Bars that do not share a letter are significantly different (*p* ≤ 0.05, Tukey’s test).

Drought negatively impacts crop productivity, leading to reduced biomass and yield. However, inoculating wheat seeds with specific strains (DTS19, DTS21, and DTS23) and biochar significantly alleviates these effects by enhancing seed germination (Figure 10a). Plants treated with these strains also exhibit improved growth parameters compared to their respective controls. In laboratory experiments, drought stress causes a 16% reduction in germination, a 21% reduction in root length, and a 10% reduction in shoot length compared to unstressed seedlings. Notably, there are significant differences in various biological parameters between wheat plants treated with drought-tolerant strains under drought stress and unstressed conditions. The drought-tolerant PGPR strains, alongside biochar (T1, T2, T3, T4, T5, T6, T7, T8, and T9), mitigate the phytotoxic effects of drought, increasing root length by 17% to 70% (Figure 10c) and shoot length by 30% to 82% (Figure 10b) compared to the inoculated controls under laboratory conditions.

As compared to the controls, the growth of wheat plants was greatly enhanced by the addition of PGPR, biochar, and biochar + PGPR. In the laboratory experiments, all treatments showed an increase in shoot fresh weight under unstressed and stressed conditions ranging from 20.2% to 69.3% and 34% to 107%, respectively, as compared to the controls (Figure 10d). The dry weight of the shoots also increased under unstressed and stressed conditions, with the increases ranging from 15% to 56% and 15% to 60%, respectively (Figure 10f). The highest increase in shoot fresh and dry weight was noted upon the application of a consortium of all strains alongside biochar under both controlled and stressed conditions. Similarly, 43.1% to 127% and 8.4% to 77.9% increases in root fresh and dry weight (Figure 10e,g) were observed under controlled conditions. Under stress conditions, root fresh and dry weight increases ranging from 13.9% to 273% and 12.9% to 113%, respectively, were achieved. Under both stressed and controlled conditions, consortiums of all strains plus biochar increased the fresh and dry weight of roots and shoots.

### 2.7. Experiment under Natural Conditions

The synergistic use of BC and PGPR strains led to notable improvements in plant growth, shoot length, chlorophyll, carotenoid levels, proline accumulation, soluble sugar content, the MSI, and relative water content (RWC) in response to induced drought stress. These improvements resulted from the synergistic interaction between the BC and PGPR strains (DTS-19, DTS-21, and DTS-23).

#### 2.7.1. Chlorophyll and Carotenoid Contents

**Figure 11:**
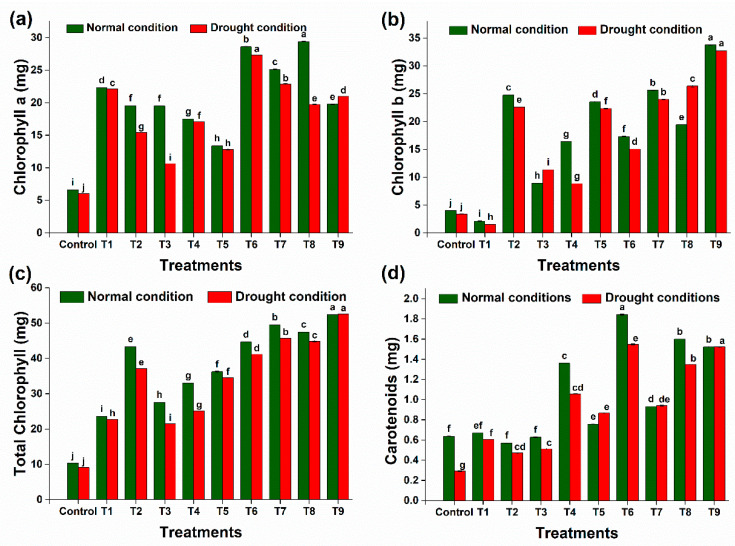
Effect of drought-tolerant rhizobacteria and biochar on biochemical parameters of plants: (**a**) chlorophyll a, (**b**) chlorophyll b, (**c**) total chlorophyll, and (**d**) carotenoid contents. Data are presented as means of 3 replicates. Bars that do not share a letter are significantly different (*p* ≤ 0.05, Tukey’s test).

The chlorophyll-a (chl-a) levels in the plants were notably diminished (14.2%) in response to drought conditions compared to the control group. Conversely, the incorporation of BC and PGPR (DTS-19, DTS-21, and DTS-23) significantly elevated chlorophyll-a levels up to 247% in the wheat leaves, surpassing those of the controls (Figure 11a). A similar pattern was observed for chlorophyll b (chl-b) content, where treatments involving biochar (BC), PGPR (DTS-19, DTS-21, and DTS-23), and their combination (BC + DTS-19 + DTS-21 + DTS-23) exhibited increased levels (240%, 566%, 235%, 160%, and 866%, respectively), while a significant decrease (25.2%) was noted under drought stress alone (Figure 11b). Furthermore, the total chlorophyll content of the plants exhibited a substantial increase (477%) under BC + DTS-19 + DTS-21 + DTS-23 treatment compared to the controls (Figure 11c). A reduction in total chlorophyll was observed under drought stress treatment alone. Carotenoid levels in the leaves increased with drought treatment, and a tremendous increase (423%) in the amount of carotenoids was observed under drought conditions upon the application of consortium + biochar (Figure 11d).

The total chlorophyll and carotenoid contents of the plants exhibited substantial increases of 477% and 423%, respectively, under BC + DTS-19 + DTS-21 + DTS-23 treatment.

#### 2.7.2. Relative Electrolyte Leakage and Relative Water Content (RWC)

Under natural conditions, electrolyte leakage in wheat increased by 13.3% when the plants were subjected to water scarcity. As compared to the untreated drought-exposed plants, the use of BC and PGPR (DTS-19, DTS-21, and DTS-23) reduced it by 19.6%, 15.6%, 15.9%, and 20.0%, respectively. Together, these treatments (BC + DTS-19 + DTS-21 + DTS-23) reduced it to 31.5%, demonstrating their synergistic impact.

**Figure 12:**
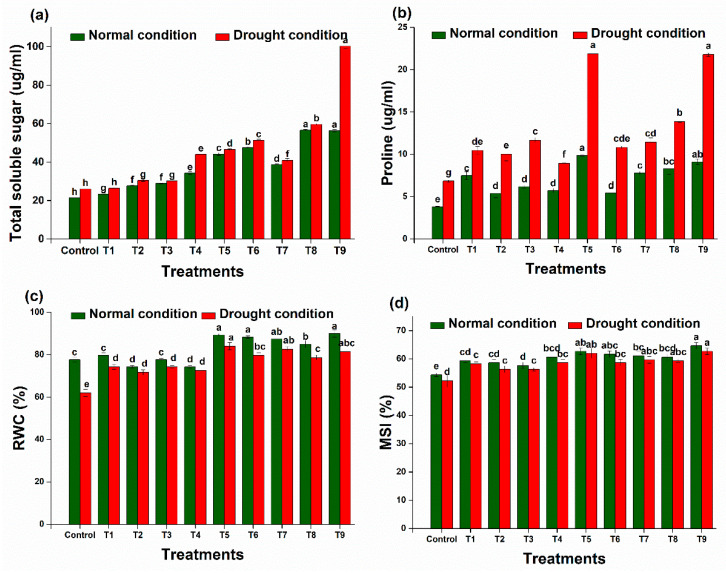
Effect of different treatments on biochemical features of wheat plants: (**a**) total soluble sugar, (**b**) proline, (**c**) relative water content (RWC), and (**d**) membrane stability index (MSI). Data are presented as means of 3 replicates. Bars that do not share a letter are significantly different (*p* ≤ 0.05, Tukey’s test).

Plants experiencing drought stress exhibited a significant decrease in water content (*p* < 0.05), as depicted in Figure 12d. In comparison to the control group, the wheat’s water content dropped by 21.1%. However, upon the application of the biochar and bacterial strains (DTS-19, DTS-21, and DTS-23) separately, there was an increase in RWC to 19.4%, 20.0%, 20.0%, and 20.2%, respectively, compared to the un-inoculated drought-stressed plants. Notably, when both biochar and bacterial strains (DTS-19, DTS-21, and DTS-23) were applied simultaneously, there was a 37.1% increase in relative water content.

#### 2.7.3. Membrane Stability Index (MSI) and Osmolyte Content

The most significant enhancements were achieved by combining biochar and bacterial strains DTS-19, DTS-21, and DTS-23, resulting in increases of 19.5% in the membrane stability index (Figure 12c). The synthesis of osmolyte (soluble sugar and proline) in wheat was prompted by drought stress. The levels of proline and total soluble sugar were higher in the plants exposed to drought compared to the well-watered ones. The use of biochar and bacterial strains DTS-19, DTS-21, and DTS-23 separately amplified sugar contents by 4%, 20.1%, 20.0%, and 72.8%, respectively. The highest increase in proline content, 219%, was observed under drought stress upon the combined use of biochar and bacterial strains DTS-19, DTS-21, and DTS-23 compared to the un-inoculated plants.

#### 2.7.4. Root Colonization

**Figure 13:**
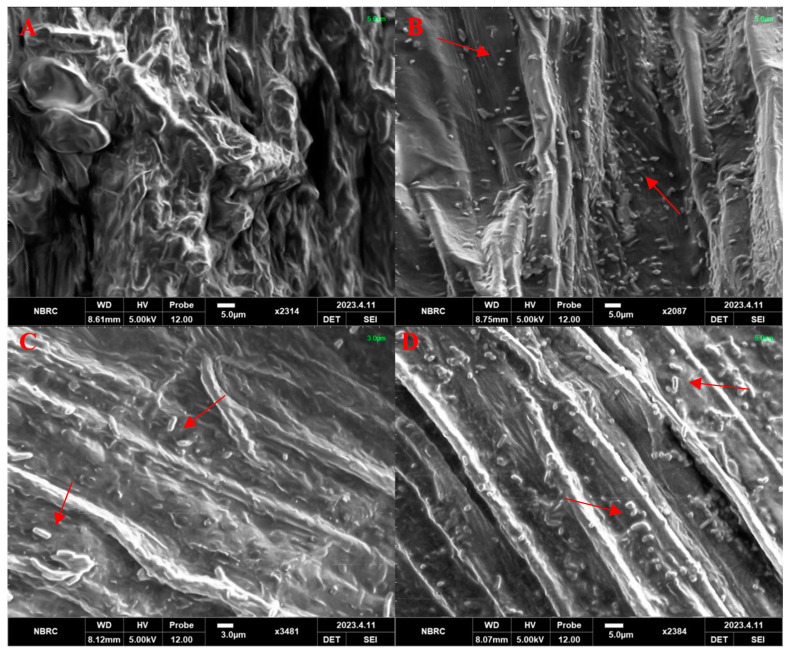
Scanning electron microscope photographs of root colonization by drought-tolerant rhizobacteria. (**A**) Un-inoculated control, (**B**) DTS-21, (**C**) DTS-19, and (**D**) DTS-23. Arrows indicate presence of bacteria on root surface.

DTS-19, DTS-21, and DTS-23 successfully colonized the roots of the wheat plants. It was observed that the PGPR made a good physical association with the root tissues. It was revealed that DTS-21 and DTS-23 were present more abundantly on the roots of wheat compared to DTS-19 when observed under a scanning electron microscope (SEM). Bacteria appeared dispersed on the root surface (Figure 13).

#### 2.7.5. Growth and Yield Components of Wheat

Without the PGPR and biochar treatments, drought stress considerably decreased the wheat growth and yield components, including shoot length, spike length, number of spikelets per spike, number of grains per spike, and 1000-grain weight (g). However, when biochar, PGPR, or both were applied to the wheat plants, the detrimental effects of drought stress were greatly reduced (Table 4).

#### 2.7.6. Nutrient Analysis of Soil

**Figure 14:**
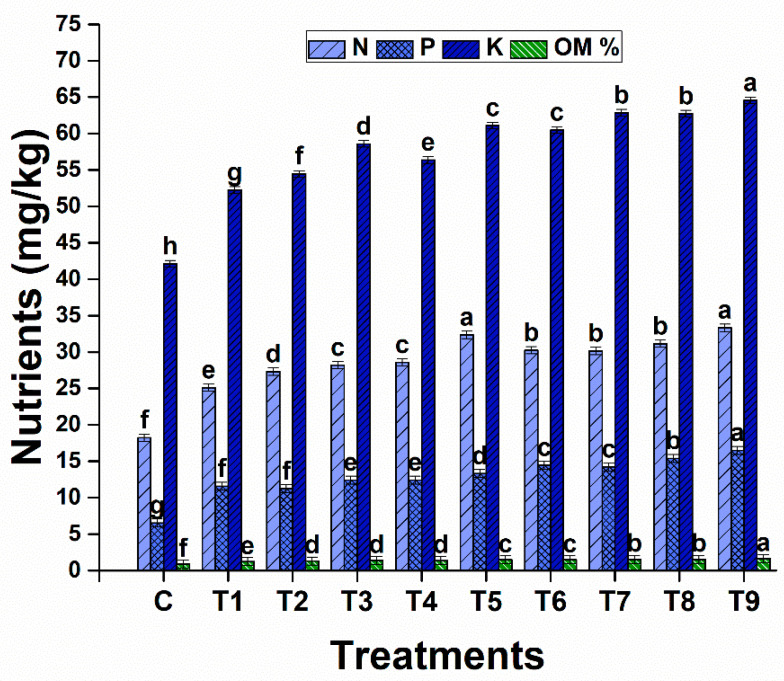
Effects of biochar and drought-tolerant rhizobacteria on available soil nitrogen, phosphorus, potassium, and organic matter content under drought stress. Data are presented as means of 3 replicates. Means that do not share a letter are significantly different (*p* ≤ 0.05, Tukey’s test).

The fertility of the soil was significantly altered with different treatments involving different combinations of biochar and PGPR. The highest concentrations of nitrogen, phosphorus, and potassium were observed in T9. The highest content of organic matter was observed in the soil of treatment 9 (Figure 14). The lowest concentration of all nutrients was noted in the stressed controls.

## 3. Discussion

Plants are constantly subjected to various abiotic stresses, and more information is required to comprehend the best ways to handle these various forms of stress. Thus, the goal of this study was to evaluate two novel approaches that may reduce the detrimental effects of water scarcity on wheat. After testing, it was discovered that using biochar and PGPR separately and in combination could be useful strategies for reducing the negative impacts of drought stress on wheat production. Our findings revealed that the use of PGPR and biochar in combination is a sustainable approach for increasing the yield of wheat in drought-affected areas.

As compared to the un-inoculated controls, the plants treated with PGPR and biochar showed improved growth and yield after improving their resilience to stress. This finding from our study is in harmony with the findings of [23]. Under drought stress, the growth of tomato plants was also improved by the application of PGPR and biochar [24].

The individual and combined application of PGPR and biochar improved the seed germination percentage. Similar findings were reported by the authors of [25]. Due to the bio-stimulation influence of PGPR and biochar, their synergistic effect increased microbial activity in the rhizosphere and further improved overall bacterial abundance. In the end, this led to improved wheat growth under stressful circumstances [26]. Compared to solitary application and the control treatment, the use of PGPR and biochar had a highly beneficial influence on physiological parameters, plant growth, and yield-related traits [21]. The production of osmolytes, increased nutrients, and the biosynthesis of phytohormones like IAA, which may be fundamentally linked to plant yield and plant components, could all be factors contributing to the improvement in plant development, as reported in [27].

Chlorophyll concentration has been proposed as a helpful indicator that can be used to evaluate physiological responses to environmental stressors like drought [28]. The primary cause of the decrease in chlorophyll under conditions of water stress is chloroplast damage [29]. This reduces agricultural yield by causing major physiological changes in plants throughout growth. When biochar and PGPR were combined, the highest levels of chlorophyll were seen together with a water deficit. This is directly tied to the amount of nitrogen in the leaves, a macronutrient needed for the synthesis of chlorophyll. These results indicated increased nitrogen uptake by leaves upon the synergistic application of biochar and PGPR [30].

Membrane stability was significantly decreased when plants were exposed to drought stress but increased upon the application of biochar and PGPR [31]. Similar results were reported by the authors of [32,33]. Osmolytes maintain osmotic potential in harsh environmental conditions to protect cells from the adverse effects of osmotic potential, as documented in [34]. PGPR increases the production of osmolytes under drought stress. Our studies showed similar results to prior studies. The genes that produce osmolytes, such as proline and sugar, are activated by PGPR.

In the current study, plants that were watered with adequate freshwater in the presence of PGPR + biochar had the highest grain yield. Wheat productivity is primarily determined by yield components such as grain weight per 1000 grains [35]. On the other hand, drought stress reduced characteristics linked to yield, limiting crop productivity. The drop in crop yield after water deficit irrigation may be due to a decrease in total soluble sugar, RWC, and total chlorophyll. In the end, this could result in a decrease in the rate of photosynthesis, a decrease in grain output, and a decrease in the harvest index [36].

Our research findings indicated that wheat yield was reduced upon exposure to drought conditions. With the application of biochar and PGPR, the adverse effects of drought were reduced, and yield was enhanced [37]. It was significantly better when these treatments were applied in combination.

## 4. Materials and Methods

### 4.1. Isolation and Purification of Rhizobacteria

Samples of rhizosphere soil were collected from diverse areas of southern Punjab, Pakistan in sterilized conditions following the protocols mentioned in [38]. The samples were taken from wheat rhizosphere growing in arid lands of district Bahawalpur (29.3544° N, 71.6911° E) and district Multan (30.1575° N, 71.5249° E), Pakistan. Rhizosphere soil was collected along the roots of growing plants in sterilized and labeled polythene bags. These bags were kept in an ice container and transported to the laboratory for rhizobacteria isolation. The physicochemical characteristics of the soil samples, such as their texture, pH, and electric conductivity, were analyzed. The isolation of bacteria was carried out by the serial dilution method [39]. On the basis of morphology, distinct colonies were selected and purified by streaking.

### 4.2. Screening of Rhizobacteria for Drought Tolerance and Morphological Characterization

The screening of drought-tolerant strains was carried out by inoculating the isolated bacterial strains in an LB broth containing different concentrations of PEG 6000, i.e., 0, 10%, 20%, 30%, and 40%. After incubating the inoculated tubes at 37 °C for 1 day, growth was measured using a spectrophotometer. Drought tolerance and sensitivity patterns were evaluated using the criteria given in [40]. A light microscope was used for morphological characterization to observe the shape, color, size, and margins of the colonies of the bacterial isolates. Gram’s reaction, cell shape, size, and the presence of endospores were also observed [41].

### 4.3. Assessment of PGP Attributes of Drought-Tolerant Rhizobacteria

An assessment of the attributes of all PGPR was performed at 40% PEG concentration.

#### 4.3.1. Screening for IAA Production

Isolated drought-tolerant bacterial isolates were screened out with the Salkowski reagent for auxin production as described in [42]. The inoculation of pure culture was carried out in LB broth. These tubes were incubated at 120 rpm in a shaker incubator at 25 °C for 1 day. Cell-free supernatant was collected by the centrifugation of incubated broth at 10,000 rpm for 10 min. The supernatant (2 mL) was collected in another test tube, and 2 mL of Salkowski’s reagent was added to each supernatant. This mixture was incubated in the dark for 60 min for color development. The appearance of a pink color indicated the production of auxin. The concentration of IAA produced was calculated at 530 nm by using a spectrophotometer. Quantification was performed by the standard graph method using pure synthetic auxin [43].

#### 4.3.2. Screening of Phosphate-Solubilizing Rhizobacteria

NBRIP media were used to check the phosphate-solubilizing ability of isolated rhizobacteria. The bacteria were inoculated on NBRIP agar media by the spot inoculation method. Plates were covered in aluminum foil and were placed in an incubator at 28 °C. The solubilizing index was calculated using the formula described in [44]. The quantification of solubilized inorganic phosphate was also carried out by the standard curve method [45].

#### 4.3.3. Screening for Nitrogen Fixation

The capability of selected rhizobacteria to fix nitrogen was estimated by growing bacteria on nitrogen-free media following the standard protocol in [46]. The bacteria were inoculated on Jensen’s medium. The appearance of the halo zone was observed after incubation at 28 °C.

#### 4.3.4. Screening for Hydrogen Cyanide Production

The hydrogen cyanide production potential of drought-tolerant PGPR was evaluated by growing them on an L-Agar plate supplemented with glycine as described in [47]. After streaking the bacteria on agar, filter paper that had been presoaked in picric acid and sodium carbonate was put over the bacteria. HCN-producing bacteria turned the filter yellow to orange upon incubation.

#### 4.3.5. Screening for Ammonia Production

For ammonia production, bacterial fresh culture was inoculated in peptone water as described in [48]. The supernatant was collected by the centrifugation of incubated peptone water. The supernatant (1 mL) and 1 mL of Nessler’s reagent were mixed. Distilled water was added to this mixture to make a total volume of 8.5 mL. The color of the mixture turned red if ammonia was produced by the bacteria.

#### 4.3.6. Screening for Production of Siderophores

Chrome Azurol S (CAS) medium was utilized to measure the sideophore production capacity of the bacterial isolates following the protocol mentioned in [38]. Bacterial cultures were streaked on CAS agar. Following a 24 h incubation period, siderophore production was confirmed when the CAS agar medium changed from blue to orange.

#### 4.3.7. Screening for Exopolysaccharide Production Potential

Drought-tolerant bacterial isolates were tested for exopolysaccharide production on modified RCV sucrose media by the protocol described in [25]. After incubation, the bacteria produced mucoid colonies when they produced exopolysaccharides. The quantification of the exopolysaccharide produced by PGPR was performed by growing bacteria in King’s B medium as described in [49].

#### 4.3.8. Screening for Production of Osmolytes

Total sugar and proline contents were evaluated by growing bacterial isolates in LB broth. The content of proline was measured in the supernatant after the centrifugation of bacteria at 1000× *g* for 10 min [50].

Total soluble sugar concentration was estimated by mixing the supernatant with anthrone reagent as described in [51]. After heating the mixture at 100× *g* centigrade for 8 min, the mixture was chilled, and optical density was noted at 630 nm. The standard curve method was used for the analysis of results.

#### 4.3.9. Screening for Extracellular Enzyme Production

Extracellular enzyme production ability, pertaining to enzymes such as pectinase, chitinase, and protease, was evaluated as described in [52]. To evaluate protease activity, bacteria were grown on skim milk agar [53]. Upon incubation, the bacteria produced a hollow zone around the colony if it produced protease. Pectinase production ability was evaluated by growing bacteria on M9 media amended with pectin [54]. A clear hollow zone appearance indicates the production of the pectinase enzyme. For our chitinase production evaluation, a colloidal solution of chitin was added to nutrient agar [55]. A clear halo zone was observed upon the production of chitinase.

### 4.4. Characterization of Biochar and Inoculum Preparation

Commercially available organic biochar made from rice husk was procured from “PLANTA nursery and garden store”. A physicochemical analysis of organic biochar was performed [56]. After drying the biochar in the oven, bacteria, 10^10^ CFU per ml, were mixed with biochar (50 g) and stored in sterilized polythene bags as bio-fertilizers. The survival rate of the bacteria in biochar was evaluated at regular intervals, i.e., 15 days, 1 month, 2 months, 3 months, 4 months, 5 months, and 6 months of incubation, by the serial dilution method as calculated with the formula used by the authors of [57].

Colony forming unit = (Number of colonies × Dilution factor)/(Volume of inoculum)

### 4.5. Treatments, Layout, and Experimental Design

Plant–microbe interaction was evaluated under both axenic and natural conditions in the winter season (November–March) in Multan. Seeds of wheat (variety Akbar-19) were procured from Punjab Seed Corporation, Multan. The Women University Multan, Multan, Pakistan, was selected for experimentation. Three PGPR bacteria, either alone or in combination with biochar, were used in these studies, together with two controls (water and stressed), and the nine treatment combinations were set up in a randomized block design with three replications.

The following treatments were tested under well-watered (90% field capacity) and stressed conditions (40% field capacity).

Control = Watered control (no PGPR, no biochar);

Control = Stressed control (no PGPR, no biochar);

T1 = Only biochar;

T2 = Only PGPR (DTS-19);

T3 = Only PGPR (DTS-21);

T4 = Only PGPR (DTS-23);

T5 = Only PGPR consortium (DTS-19 + DTS-21+ DTS-23);

T6 = PGPR (DTS-19) + biochar;

T7 = PGPR (DTS-21) + biochar;

T8 = PGPR (DTS-23) + biochar;

T9 = PGPR consortium (DTS-19 + DTS-21+ DTS-23) + biochar.

### 4.6. Wheat Growth Promotion under Axenic and Natural Conditions

Three selected bacterial isolates, alongside biochar, were used as carrier materials for the alleviation of drought stress in wheat in experiments. Axenic conditions were maintained in the laboratory experiments. Nine experimental sets of seeds (watered controls, stressed controls, with and without PGPR inoculation, and with and without biochar inoculation) were used. For irrigation purposes, a 10% PEG solution was used for the stress conditions. Autoclaved distilled water was applied for the water control treatment. Each treatment was run in replicates of 3. A total of 60 plastic pots were used for this experiment with RCBD. Seeds of wheat, surface sterilized with 0.1% mercuric chloride followed by washing with autoclaved distilled water, were coated with an inoculated biochar carrier mixed with carboxy methyl cellulose (CMC). For the un-inoculated controls, seeds were coated with sterilized carriers using the same procedure. Liquid inoculum of strains without carrier material was also tested by dipping seeds in liquid inoculum for 30 min. Autoclaved soil (200 g) was added in plastic cups (3.6″ × 2.7″). The coated seeds (10 seeds per pot) were sown in pots. The pots were placed in a growth chamber with a 12 h photoperiod (light intensity of 2200 lux) regime at 28 ± 2 °C for 14 days. After germination, different physiomorphological characteristics (root length, shoot length, and fresh and dry weight of roots and shoots) were measured.

After the evaluation of plant–microbe interactions in axenic conditions, another experiment on the above-mentioned seed treatments was carried out under natural conditions. Under natural conditions, experimentation was performed in earthen pots at 40% field capacity. Two kinds of controls (un-inoculated unstressed and un-inoculated stressed controls) were run during the experiment. A total of 60 pots were used in this experiment. Autoclaved soil (10 kg) was added in earthen pots. Coated seeds (10 seeds per pot) were sown in the pots. After 21 days of germination, stress was applied, and leaves were sampled for the measurement of physicochemical parameters (chlorophyll content, RWC, REL, proline content, membrane stability, and total soluble sugar content). For the evaluation of yield parameters, plants from 3 pots of each treatment were harvested after 120 days of sowing.

### 4.7. Physiological Parameters

#### 4.7.1. Carotenoid and Chlorophyll Contents

The carotenoid and chlorophyll contents of the stressed inoculated and control stressed plants were measured in acetone (80%) as described in [58]. For this purpose, a 0.1 g fresh leave sample from each treatment was taken and incubated in 80% acetone (10 mL) for 24 h. After overnight incubation, the material was centrifuged at 5000 rpm for 0.5 min. We collected the supernatant and recorded the absorbance of each supernatant at 663 nm, 645 nm, and 470 nm.

#### 4.7.2. Relative Water Content (RWC)

For RWC, leaf samples from all treatments were obtained and weighed (FW) immediately as described in [59]. Then, each leaf sample was immersed in double-distilled water for 1 day (24 h) at 25 °C. Then, after 24 h, turgid weight (TW) was measured for each treatment. Then, the leaves were placed in an oven to measure dry weight (DW). The following formula was used to calculate RWC [60].

% RWC = {FW − DW/TW − DW} × 100

#### 4.7.3. Relative Electrolyte Leakage

For relative electrolyte leakage (REL), half a gram of fresh leaf sample was taken from all treatments. Leaf discs were cut and dipped in double-distilled water for 4 h. After four hours, the electrolyte conductivity of each sample was measured (EC1). After EC1 measurement, this solution was autoclaved for half an hour. Then, it was allowed to cool, and electrolyte conductivity was again measured (EC2). The following formula was used to calculate REL as described in [61].

% REL = C1/C2 × 100

#### 4.7.4. Membrane Stability Index (MSI)

For the MSI, 0.1 g fresh leaf samples in the form of discs were obtained [62]. The discs were washed with double-distilled water. After placing these discs in 10 mL double-distilled water in test tubes, the test tubes were heated for half an hour at 40 °C. After half an hour, electrolyte conductivity was calculated (C1). Then, these same test tubes were placed in a water bath again at 100 °C for 10 min. The tubes were cooled, and electrolyte conductivity was again noted (C2). The following formula was used to calculate the MSI as described in [63].

Membrane stability index (MSI) = {1 − (C1/C2)} × 100

### 4.8. Biochemical Parameters

#### 4.8.1. Proline Content

Proline accumulation in the leaves was determined as explained in [64]. For proline estimation, 0.5 g leaf samples from each treatment were taken and homogenized in 10 of 3% sulfosalicylic acid. Using Whatman filter paper no.2, homogenate was filtered. Next, 2 mL of filtrate was mixed with the same amount of acid ninhydrin and glacial acetic acid. The mixture was boiled at 100 °C for 60 min. After 60 min, an ice bath was used to stop the reaction. Toluene (4 mL) was added to this mixture for extraction. The reaction mixture was vigorously shaken for 30 s, and the absorbance of the extract was recorded at 520 nm. Toluene was used as a blank. The standard curve method was used to calculate proline content.

#### 4.8.2. Total Soluble Sugar Content

The content of total soluble sugar in the wheat leaves was measured using the protocol described in [65]. For this, 0.1 g leaf samples from all treatments were obtained and added to test tubes in 5 mL of ethanol (80%). A water bath was used to heat this mixture at 80 °C. After 60 min, 1 mL of this reaction mixture was mixed with 1 mL 18% phenol and 1 mL distilled water, and the mixture was then left to stand for 60 min at room temperature. After 1 h, 5 mL sulfuric acid was added to this mixture and vortexed. Then, we recorded the optical density of the mixture at a 490 nm wavelength using a spectrophotometer. Ethanol (80%) was used as a blank.

### 4.9. Root Colonization

A scanning electron microscope (SEM) was used to visualize bacterial colonization [66]. After carefully washing the root samples with sterile saline solution to get rid of any soil particles, they were fixed in 2.5% glutaraldehyde. They were again fixed for another 120 min in the same buffer using 2% aqueous osmium trioxide. Samples were dehydrated in series using graded alcohol post-fixation. An automated sputter coater was used to coat the dried samples with gold for approximately three minutes while they were mounted on stubs. Lastly, the samples were examined at different magnifications using a scanning electron microscope (Model: SEM Cube 10, Emcraft South Korea, Kwangju, Republic of Korea).

### 4.10. Yield Parameters

The wheat yield was measured in terms of shoot length, spike length, no. of spikelets per spike, number of grains per spike, and 1000-grain weight as described in [67].

### 4.11. Nutrient Analysis of Soil

After harvesting the plants, soil from the pots was collected and analyzed in terms of the available nitrogen, phosphorus, and potassium concentrations in the soil. The organic matter in the soil was also determined [68].

### 4.12. Statistical Analysis

Minitab software version number 17 was used for statistical analysis. A one-way ANOVA and Tukey’s test (the significance levels used for the tests were *p* < 0.05) were used to compare means and to determine significant differences between the treatments [69].

## 5. Conclusions

The current study has shown that the combined application of biochar and PGPR under drought stress is a sustainable approach for improving wheat growth and wheat yield. PGPR mitigated the harmful effects of drought stress by increasing seed germination and improving plant growth. They provide plants with plant hormones and improve RWC and the MSI. They also increased the concentrations of chl-a, chl-b, carotenoids, and total chlorophyll in the plants. Osmotic pressure was balanced by the production and accumulation of osmolytes such as proline and soluble sugar. Thus, it is concluded that the application of biochar and PGPR alleviated the negative effects of drought stress and enhanced the growth of wheat under drought stress.

Economic aspects related to the cost of preparing biochar and PGPR should also be studied in the future.

## Figures and Tables

**Table 1:** Plant growth-promoting attributes of drought-tolerant bacterial isolates.

Bacterial Isolates	Phosphate Solubilization (μg/mL)	EPS Production (μg/mL)	Auxin Synthesis (μg/mL)		Nitrogen Fixation	HCN Production	Ammonia Production
			Without L-Tryp	With 0.1% L-Tryp			
DTS-1	397 ± 0.64	55.3 ± 0.25	7.80 ± 0.51	56.2 ± 0.61	-	₊	-
DTS-2	235 ± 0.58	27.2 ±1.35	11.6 ± 0.53	95.6 ± 0.31	-	₊	-
DTS-3	NS	65.2 ± 0.93	6.70 ± 0.21	55.7 ± 0.21	₊	-	₊
DTS-4	NS	46.8 ± 1.48	10.9 ± 0.62	85.7 ± 0.21	-	-	-
DTS-5	235 ± 0.53	11.7 ± 0.55	10.3 ± 0.66	75.4 ± 0.68	₊	₊	₊
DTS-6	431 ± 0.98	41.9 ± 0.51	13.9 ± 0.35	114 ± 0.2	₊	₊	₊
DTS-7	NS	27.3 ± 0.47	23.5 ± 0.51	320 ± 0.6	₊	-	₊
DTS-8	NS	25.2 ± 0.35	28.6 ± 0.57	244 ± 0.2	-	₊₊	-
DTS-9	329 ± 1.03	31.8 ± 0.42	5.70 ± 0.55	51.1 ± 0.02	-	-	-
DTS-10	161 ± 0.63	64.8 ± 0.45	8.50 ± 0.33	70.0 ± 0.21	₊	-	₊
DTS-11	NS	21.8 ± 0.30	7.30 ± 0.23	53.9 ± 0.20	₊	-	₊
DTS-12	117 ± 0.46	77.5 ± 0.75	16.3 ± 0.52	172 ± 0.2	-	₊	-
DTS-13	188 ±1.04	42.8 ± 0.35	7.60 ± 0.32	54.0 ± 0.40	-	₊	-
DTS-14	NS	52.8 ± 0.41	8.10 ± 0.65	70.8 ± 0.31	₊	₊₊	₊
DTS-15	213 ± 0.93	49.8 ± 0.60	13.4 ± 0.43	111 ± 0.2	-	-	-
DTS-16	00.0 ± 0.00	58.8 ± 0.40	34. ± 0.21	99.7 ± 0.36	₊₊	-	₊₊
DTS-17	217 ± 0.57	32.7 ± 0.65	13.6 ± 0.65	125 ± 0.5	-	-	-
DTS-18	360 ± 0.66	71.6 ±1.45	11.5 ± 0.51	456 ± 0.5	-	₊	-
DTS-19	NS	23.8 ± 0.83	44.1 ± 0.42	84.0 ± 0.76	₊	₊₊	₊
DTS-20	221 ± 0.51	44.0 ± 0.55	14.3 ± 0.53	115 ± 0.2	-	₊	-
DTS-21	415 ± 0.58	81.0 ± 0.70	41.7 ± 0.31	458 ± 0.2	₊₊	₊₊₊	₊₊
DTS-22	163 ±1.15	70.3 ± 0.26	46.8 ± 0.55	43.7 ± 0.26	₊₊	₊₊₊	₊₊
DTS-23	899 ± 0.44	77.5 ± 0.76	6.20 ± 0.33	443 ± 0.2	₊	-	₊

All values are the mean of three replications ± SD. NS means not solubilizing phosphate. “+” indicates presence of activity. “-” indicates absence of activity. “+”, “++” and “+++” show intensity of the activity.

**Table 2:** Physicochemical characteristics of rice husk biochar.

Parameter	Value
Ash content	15.8%
Moisture content	3.12%
pH	7.8
EC (ds^−1^)	2.91
Total N%	1.98 ± 0.02
Total phosphorus (g/kg)	2.01 ± 0.02
Total potassium (g/kg)	2.07 ± 0.03

**Table 3:** Bacterial population density (1 × 10^8^ CFU g^−1^) in biochar carrier at room temperature.

Bacterial Isolates	Incubation (Days)						
	15	30	60	90	120	150	180
DTS-19	29.3 ± 0.5 ^c^	29.6 ± 0.5 ^c^	28.0 ± 2.6 ^b^	21.3 ± 2.0 ^bc^	17.3 ± 0.5 ^b^	7.6 ± 0.5 ^c^	2.3 ± 0.5 ^bc^
DTS-21	31.6 ± 0.5 ^b^	30.3 ± 0.5 ^b^	26.3 ± 1.5 ^c^	22.3 ± 0.5 ^b^	15.3 ± 2.5 ^c^	9 ± 1.00 ^b^	3.6 ± 1.5 ^b^
DTS-23	35.3 ± 1.5 ^a^	34.6 ± 0.5 ^a^	34.3 ± 1.5 ^a^	28.3 ± 0.5 ^a^	22.6 ± 2.5 ^a^	14.3 ± 1.1 ^a^	12.3 ± 2.5 ^a^

All values are the means of three replications ± standard deviation. Values that do not share a letter are significantly different (*p* ≤ 0.05, Tukey’s test).

**Table 4:** Effects of different treatments on yield and its parameters under watering and drought conditions.

Treatments	Shoot Length (cm)	Spike Length (cm)	No. of Spikelet per Spike	No. of Grains per Spike	Weight of 1000 Grains	Shoot Length (cm)	Spike Length (cm)	No. of Spikelet per Spike	No. of Grains per Spike	Weight of 1000 Grains
	Unstressed					Stressed				
Control	62.33 ± 3.78 ^e^	5.33 ± 0.55 ^c^	7.0 ± 0.1 ^d^	21.0 ± 0.1 ^c^	34.66 ± 0.57 ^e^	56 ± 2.0 ^d^	5.11 ± 0.57 ^d^	5.94 ± 0.57 ^b^	16 ± 1.73 ^b^	31.3 ± 1.52 ^e^
T1	72 ± 3.46 ^d^	7 ± 1.0 ^bc^	12.33 ± 0.57 ^c^	37 ± 1.73 ^b^	39.6 ± 1.15 ^cd^	71.3 ± 4.72 ^bc^	6.67 ± 1 ^bc^	11.3 ± 1.52 ^a^	40 ± 4.58 ^a^	37 ± 1 ^bc^
T2	74.3 ± 4.04 ^cd^	8.1 ± 1 ^ab^	13.3 ± 0.57 ^bc^	40 ± 1.73 ^ab^	37.3 ± 0.57 ^de^	73.3 ± 2.08 ^bc^	7.66 ± 1.52 ^ab^	12 ± 1 ^a^	39 ± 3 ^a^	34.6 ± 0.57 ^de^
T3	73.3 ± 2.30 ^d^	8.66 ± 0.57 ^ab^	14.2 ± 0 ^bc^	42.1 ± 0 ^ab^	40.3 ± 1.17 ^bcd^	72 ± 4 ^bc^	6.66 ± 0.57 ^bc^	12.6 ± 1.52 ^a^	38 ± 4.58 ^a^	38 ± 3 ^bcd^
T4	76.3 ± 1.53 ^bcd^	7.33 ± 0.57 ^bc^	13.6 ± 0.57 ^bcd^	41 ± 1.73 ^abd^	41.3 ± 0.5 ^bc^	73 ± 1 ^bc^	7.13 ± 0.57 ^bc^	11.6 ± 0.57 ^a^	35 ± 1.73 ^a^	40.3 ± 0.57 ^abc^
T5	78.6 ± 1.52 ^cd^	8.1 ± 0 b^ab^	14.53 ± 2.08 ^bc^	43 ± 6.24 ^ab^	44 ± 1.0 ^ab^	74 ± 1 ^bc^	6.3 ± 0.57 ^c^	12.6 ± 2.08 ^a^	38 ± 6.24 ^a^	41 ± 1.2 ^abc^
T6	81.3 ± 3.51 ^abc^	9.33 ± 1.15 ^ab^	14.33 ± 0.57 ^bc^	43 ± 1.73 ^ab^	41.6 ± 2.0 ^bc^	75.3 ± 0.57 ^bc^	8.18 ± 0.55 ^a^	12.6 ± 0.55 ^a^	37 ± 1.73 ^a^	38.3 ± 3.05 ^abcd^
T7	84 ± 2 ^ab^	9.33 ± 0.57 ^ab^	14.3 ± 0.57 ^bc^	42 ± 1.73 ^ab^	43.3 ± 0.5 ^abc^	76 ± 1 ^b^	7.9 ± 0.5 ^ab^	12.6 ± 0.57 ^a^	38 ± 1.73 ^a^	42 ± 0 ^abc^
T8	85.6 ± 0.57 ^a^	9.6 ± 0.57 ^a^	15.1 ± 0 ^b^	45 ± 0 ^ab^	43.66 ± 2.5 ^ab^	78.6 ± 0.57 ^b^	8.3 ± 0.57 ^a^	13.1 ± 0.57 ^a^	40 ± 1.73 ^a^	43.3 ± 1.5 ^a^
T9	86.6 ± 1.52 ^ab^	11 ± 0 ^a^	16.6 ± 0.57 ^a^	48 ± 1.73 ^a^	45.66 ± 1.52 ^a^	81 ± 1.73 ^a^	8.2 ± 0.57 ^a^	15 ± 0 ^a^	45 ± 0 ^a^	43.3 ± 1.53 ^a^

Data are presented as means + standard deviations of 3 replicates. Means that do not share a letter are significantly different (*p* ≤ 0.05, Tukey’s test).

## Data Availability

The original contributions related to the study are included in the article; further inquiries can be directed to the corresponding author.

## References

[1] Mahreen N., Yasmin S., Asif M., Yahya M., Ejaz K., Yousaf S., Amin I., Zulfiqar S., Imran A., Khaliq S. (2023). Mitigation of water scarcity with sustained growth of Rice by plant growth promoting bacteria. Front. Plant Sci..

[2] Rana N., Bansal R., Sharma S., Sharma Y., Sonah H., Deshmukh R., Sharma T.R. (2020). Global perspectives on agriculture: Food security and nutrition. Advances in Agri-Food Biotechnology.

[3] Raza M.A.S., Zulfiqar B., Iqbal R., Muzamil M.N., Aslam M.U., Muhammad F., Amin J., Aslam H.M.U., Ibrahim M.A., Uzair M. (2023). Morpho-physiological and biochemical response of wheat to various treatments of silicon nano-particles under drought stress conditions. Sci. Rep..

[4] Raza M.A.S., Saleem A., Khan I.H., Tahir M.A., Iqbal R., Aslam M.U., Alamoudi S.A., Ali B., Javed M.A., Al Syaad K.M. (2023). Amelioration of Drought Stress in Wheat by Using Plant Growth Promoting Rhizobacteria (PGPR) and Biogas Slurry. Res. Sq..

[5] Uzma M., Iqbal A., Hasnain S. (2022). Drought tolerance induction and growth promotion by indole acetic acid producing Pseudomonas aeruginosa in Vigna radiata. PLoS ONE.

[6] Hussein H.-A.A., Alshammari S.O., Kenawy S.K., Elkady F.M., Badawy A.A. (2022). Grain-priming with L-arginine improves the growth performance of wheat (*Triticum aestivum* L.) plants under drought stress. Plants.

[7] Ahmad A., Aslam Z., Hussain S., Javed T., Hussain S., Bashir S., Hussain I., Belliturk K., Adamski R., Siuta D. (2022). Soil application of wheat straw vermicompost enhances morpho-physiological attributes and antioxidant defense in wheat under drought stress. Front. Environ. Sci..

[8] Amini A., Majidi M.M., Mokhtari N., Ghanavati M. (2023). Drought stress memory in a germplasm of synthetic and common wheat: Antioxidant system, physiological and morphological consequences. Sci. Rep..

[9] Bhanbhro N., Wang H.-J., Yang H., Xu X.-J., Jakhar A.M., Zhang R.-X., Bakhsh Q., Akbar G., Jakhro M.I., Chen K.-M. (2023). Revisiting the molecular mechanisms and adaptive strategies associated with drought stress tolerance in common wheat (*Triticum aestivum* L.). Plant Stress.

[10] Wang W., Vinocur B., Altman A. (2003). Plant responses to drought, salinity and extreme temperatures: Towards genetic engineering for stress tolerance. Planta.

[11] Van Esse H.P., Reuber T.L., van der Does D. (2020). Genetic modification to improve disease resistance in crops. New Phytol..

[12] Zaib S., Zubair A., Abbas S., Hussain J., Ahmad I., Shakeel S.N. (2023). Plant Growth-Promoting Rhizobacteria (PGPR) Reduce Adverse Effects of Salinity and Drought Stresses by Regulating Nutritional Profile of Barley. Appl. Environ. Soil Sci..

[13] Bouremani N., Cherif-Silini H., Silini A., Bouket A.C., Luptakova L., Alenezi F.N., Baranov O., Belbahri L. (2023). Plant growth-promoting rhizobacteria (PGPR): A rampart against the adverse effects of drought stress. Water.

[14] Ferioun M., Srhiouar N., Tirry N., Belahcen D., Siang T.C., Louahlia S., El Ghachtouli N. (2023). Optimized drought tolerance in barley (*Hordeum vulgare* L.) using plant growth-promoting rhizobacteria (PGPR). Biocatal. Agric. Biotechnol..

[15] Gowtham H.G., Singh S.B., Shilpa N., Aiyaz M., Nataraj K., Udayashankar A.C., Amruthesh K.N., Murali M., Poczai P., Gafur A. (2022). Insight into recent progress and perspectives in improvement of antioxidant machinery upon PGPR augmentation in plants under drought stress: A review. Antioxidants.

[16] Ansari F.A., Ahmad I., Pichtel J. (2023). Synergistic effects of biofilm-producing PGPR strains on wheat plant colonization, growth and soil resilience under drought stress. Saudi J. Biol. Sci..

[17] Saengsanga T., Phakratok N., Rattana T. (2023). Bioformulations Derived from *Enterobacter* sp. NRRU-N13 and Oligochitosan Alleviate Drought Stress in Thai Jasmine Rice (*Oryza sativa* L. var. KDML105). Microbes Environ..

[18] Safdar H., Jamil M., Hussain A., Albalawi B.F.A., Ditta A., Dar A., Aimen A., Ahmad H.T., Nazir Q., Ahmad M. (2022). The Effect of Different Carrier Materials on the Growth and Yield of Spinach under Pot and Field Experimental Conditions. Sustainability.

[19] Perveen R., Hussain A., Ditta A., Dar A., Aimen A., Ahmad M., Alataway A., Dewidar A.Z., Mattar M.A. (2023). Growth and yield of Okra exposed to a consortium of rhizobacteria with different organic carriers under controlled and natural field conditions. Horticulturae.

[20] Racioppi M., Tartaglia M., De la Rosa J.M., Marra M., Lopez-Capel E., Rocco M. (2019). Response of ancient and modern wheat varieties to biochar application: Effect on hormone and gene expression involved in germination and growth. Agronomy.

[21] Zulfiqar B., Raza M.A.S., Saleem M.F., Aslam M.U., Iqbal R., Muhammad F., Amin J., Ibrahim M.A., Khan I.H. (2022). Biochar enhances wheat crop productivity by mitigating the effects of drought: Insights into physiological and antioxidant defense mechanisms. PLoS ONE.

[22] Omara A.E.-D., Hafez E.M., Osman H.S., Rashwan E., El-Said M.A., Alharbi K., Abd El-Moneim D., Gowayed S.M. (2022). Collaborative impact of compost and beneficial rhizobacteria on soil properties, physiological attributes, and productivity of wheat subjected to deficit irrigation in salt affected soil. Plants.

[23] Khan Z., Khan M.N., Zhang K., Luo T., Zhu K., Hu L. (2021). The application of biochar alleviated the adverse effects of drought on the growth, physiology, yield and quality of rapeseed through regulation of soil status and nutrients availability. Ind. Crops Prod..

[24] Yuan W., Wenqing L., Binghai D., Hanhao L. (2021). Effect of biochar applied with plant growth-promoting rhizobacteria (PGPR) on soil microbial community composition and nitrogen utilization in tomato. Pedosphere.

[25] Ilyas N., Mumtaz K., Akhtar N., Yasmin H., Sayyed R., Khan W., Enshasy H.A.E., Dailin D.J., Elsayed E.A., Ali Z. (2020). Exopolysaccharides producing bacteria for the amelioration of drought stress in wheat. Sustainability.

[26] Siddikee M.A., Chauhan P.S., Anandham R., Han G.-H., Sa T.-M. (2010). Isolation, characterization, and use for plant growth promotion under salt stress, of ACC deaminase-producing halotolerant bacteria derived from coastal soil. J. Microbiol. Biotechnol..

[27] Ijaz M., Tahir M., Shahid M., Ul-Allah S., Sattar A., Sher A., Mahmood K., Hussain M. (2019). Combined application of biochar and PGPR consortia for sustainable production of wheat under semiarid conditions with a reduced dose of synthetic fertilizer. Braz. J. Microbiol..

[28] Gharred J., Derbali W., Derbali I., Badri M., Abdelly C., Slama I., Koyro H.-W. (2022). Impact of biochar application at water shortage on biochemical and physiological processes in medicago ciliaris. Plants.

[29] Pandit N.R., Schmidt H.P., Mulder J., Hale S.E., Husson O., Cornelissen G. (2019). Nutrient effect of various composting methods with and without biochar on soil fertility and maize growth. Arch. Agron. Soil Sci..

[30] Chandra D., Srivastava R., Glick B.R., Sharma A.K. (2018). Drought-tolerant Pseudomonas spp. improve the growth performance of finger millet (*Eleusine coracana* (L.) Gaertn.) under non-stressed and drought-stressed conditions. Pedosphere.

[31] Ansari F., Jabeen M., Ahmad I. (2021). Pseudomonas azotoformans FAP5, a novel biofilm-forming PGPR strain, alleviates drought stress in wheat plant. Int. J. Environ. Sci. Technol..

[32] Hussain M.B., Zahir Z.A., Mehboob I., Mahmood S., Ahmed N., Haq T.U., Ahmad I., Imran M. (2019). Mesorhizobium ciceri-CR-39 inoculation to wheat for drought tolerance at critical growth stages. Pak. J. Bot..

[33] Liu F., Ma H., Liu B., Du Z., Ma B., Jing D. (2023). Effects of plant growth-promoting rhizobacteria on the physioecological characteristics and growth of walnut seedlings under drought stress. Agronomy.

[34] Li H., Guo Q., Jing Y., Liu Z., Zheng Z., Sun Y., Xue Q., Lai H. (2020). Application of Streptomyces pactum Act12 enhances drought resistance in wheat. J. Plant Growth Regul..

[35] Çığ F., Sönmez F., Nadeem M.A., Sabagh A.E. (2021). Effect of biochar and PGPR on the growth and nutrients content of einkorn wheat (*Triticum monococcum* L.) and post-harvest soil properties. Agronomy.

[36] Zhang J., Zhang S., Cheng M., Jiang H., Zhang X., Peng C., Lu X., Zhang M., Jin J. (2018). Effect of drought on agronomic traits of rice and wheat: A meta-analysis. Int. J. Environ. Res. Public Health.

[37] Raza M.A.S., Haider I., Farrukh Saleem M., Iqbal R., Usman Aslam M., Ahmad S., Abbasi S.H. (2021). Integrating biochar, rhizobacteria and silicon for strenuous productivity of drought-stressed wheat. Commun. Soil Sci. Plant Anal..

[38] Ashry N.M., Alaidaroos B.A., Mohamed S.A., Badr O.A., El-Saadony M.T., Esmael A. (2022). Utilization of drought-tolerant bacterial strains isolated from harsh soils as a plant growth-promoting rhizobacteria (PGPR). Saudi J. Biol. Sci..

[39] Li J., Zhou B., Li T., Lin H., Lin Z., Lu G., Liu Y., Lin B., Lin D. (2023). Isolation of rhizobacteria from the Cenchrus fungigraminus rhizosphere and characterization of their nitrogen-fixing performance and potential role in plant growth promotion. Plant Soil.

[40] Sukorini H., Putri T., Retno E., Ishartati E., Sufianto S., Setyobudi R.H., Huu N.N., Suwannarat S. (2023). Assessment on Drought Stress Resistance, Salinity Endurance, and Indole Acetic Acid Production Potential of Dryland-Isolated Bacteria. Jordan J. Biol. Sci..

[41] Alshehrei F.M. (2023). Isolation and Identification of Microorganisms associated with high-quality and low-quality cosmetics from different brands in Mecca region-Saudi Arabia. Saudi J. Biol. Sci..

[42] Khan N., Ali S., Tariq H., Latif S., Yasmin H., Mehmood A., Shahid M.A. (2020). Water conservation and plant survival strategies of rhizobacteria under drought stress. Agronomy.

[43] Saleem S., Iqbal A., Ahmed F., Ahmad M. (2021). Phytobeneficial and salt stress mitigating efficacy of IAA producing salt tolerant strains in Gossypium hirsutum. Saudi J. Biol. Sci..

[44] Zolfaghari R., Rezaei K., Fayyaz P., Naghiha R., Namvar Z. (2021). The effect of indigenous phosphate-solubilizing bacteria on Quercus brantii seedlings under water stress. J. Sustain. For..

[45] Chawngthu L., Hnamte R., Lalfakzuala R. (2020). Isolation and characterization of rhizospheric phosphate solubilizing bacteria from wetland paddy field of Mizoram, India. Geomicrobiol. J..

[46] Lotfi N., Soleimani A., Çakmakçı R., Vahdati K., Mohammadi P. (2022). Characterization of plant growth-promoting rhizobacteria (PGPR) in Persian walnut associated with drought stress tolerance. Sci. Rep..

[47] Sandhya V., Shrivastava M., Ali S.Z., Sai Shiva Krishna Prasad V. (2017). Endophytes from maize with plant growth promotion and biocontrol activity under drought stress. Russ. Agric. Sci..

[48] Chieb M., Gachomo E.W. (2023). The role of plant growth promoting rhizobacteria in plant drought stress responses. BMC Plant Biol..

[49] Latif M., Bukhari S.A.H., Alrajhi A.A., Alotaibi F.S., Ahmad M., Shahzad A.N., Dewidar A.Z., Mattar M.A. (2022). Inducing drought tolerance in wheat through exopolysaccharide-producing rhizobacteria. Agronomy.

[50] Bates L.S., Waldren R., Teare I. (1973). Rapid determination of free proline for water-stress studies. Plant Soil.

[51] Upadhyay S., Singh J., Singh D. (2011). Exopolysaccharide-producing plant growth-promoting rhizobacteria under salinity condition. Pedosphere.

[52] Moustaine M., Elkahkahi R., Benbouazza A., Benkirane R., Achbani E. (2017). Effect of plant growth promoting rhizobacterial (PGPR) inoculation on growth in tomato (*Solanum lycopersicum* L.) and characterization for direct PGP abilities in Morocco. Int. J. Environ. Agric. Biotechnol..

[53] Ghodsalavi B., Ahmadzadeh M., Soleimani M., Madloo P.B., Taghizad-Farid R. (2013). Isolation and characterization of rhizobacteria and their effects on root extracts of Valeriana officinalis. Aust. J. Crop Sci..

[54] Rhizobacteria P.G.-P. (2014). Isolation and characterization of rhizobia and plant growth-promoting rhizobacteria and their effects on growth of rice seedlings. Am. J. Agric. Biol. Sci..

[55] Pandey A.K., Dinesh K., Yadav S., Sharma H.K., Babu A. (2023). Functional traits and phylogenetic analysis of top-soil inhabiting rhizobacteria associated with tea rhizospheres in North Bengal, India. Curr. Res. Microb. Sci..

[56] Krishnananthy R., Premanandarajah P. The effect of plant growth promoting rhizobacteria [pgpr] with biochar and chemical fertilizers on soil physicochemical properties and root growth of maize grown in sandy regosol. Proceedings of the 27th Annual Technological Advances in Science, Medicine and Engineering Conference.

[57] Ullah S., Shah S., Jamal A., Saeed M.F., Mihoub A., Zia A., Ahmed I., Seleiman M.F., Mancinelli R., Radicetti E. (2024). Combined Effect of Biochar and Plant Growth-Promoting Rhizbacteria on Physiological Responses of Canola (*Brassica napus* L.) Subjected to Drought Stress. J. Plant Growth Regul..

[58] Ahmed T., Noman M., Manzoor N., Shahid M., Hussaini K.M., Rizwan M., Ali S., Maqsood A., Li B. (2021). Green magnesium oxide nanoparticles-based modulation of cellular oxidative repair mechanisms to reduce arsenic uptake and translocation in rice (*Oryza sativa* L.) plants. Environ. Pollut..

[59] Batool T., Ali S., Seleiman M.F., Naveed N.H., Ali A., Ahmed K., Abid M., Rizwan M., Shahid M.R., Alotaibi M. (2020). Plant growth promoting rhizobacteria alleviates drought stress in potato in response to suppressive oxidative stress and antioxidant enzymes activities. Sci. Rep..

[60] Slimani A., Raklami A., Oufdou K., Meddich A. (2023). Isolation and characterization of PGPR and their potenzial for drought alleviation in barley plants. Gesunde Pflanz..

[61] Zafar-ul-Hye M., Akbar M.N., Iftikhar Y., Abbas M., Zahid A., Fahad S., Datta R., Ali M., Elgorban A.M., Ansari M.J. (2021). Rhizobacteria inoculation and caffeic acid alleviated drought stress in lentil plants. Sustainability.

[62] Mansour E., Mahgoub H.A., Mahgoub S.A., El-Sobky E.-S.E., Abdul-Hamid M.I., Kamara M.M., AbuQamar S.F., El-Tarabily K.A., Desoky E.-S.M. (2021). Enhancement of drought tolerance in diverse Vicia faba cultivars by inoculation with plant growth-promoting rhizobacteria under newly reclaimed soil conditions. Sci. Rep..

[63] Shankar A., Prasad V. (2023). Potential of desiccation-tolerant plant growth-promoting rhizobacteria in growth augmentation of wheat (*Triticum aestivum* L.) under drought stress. Front. Microbiol..

[64] Khan N., Bano A., Rahman M.A., Guo J., Kang Z., Babar M.A. (2019). Comparative physiological and metabolic analysis reveals a complex mechanism involved in drought tolerance in chickpea (*Cicer arietinum* L.) induced by PGPR and PGRs. Sci. Rep..

[65] Ghosh D., Gupta A., Mohapatra S. (2019). A comparative analysis of exopolysaccharide and phytohormone secretions by four drought-tolerant rhizobacterial strains and their impact on osmotic-stress mitigation in Arabidopsis thaliana. World J. Microbiol. Biotechnol..

[66] Ansari F.A., Ahmad I., Pichtel J., Husain F.M. (2024). Pantoea agglomerans FAP10: A novel biofilm-producing PGPR strain improves wheat growth and soil resilience under salinity stress. Environ. Exp. Bot..

[67] Rostamian A., Moaveni P., Mozafari H., Rajabzadeh F. (2023). Effective drought mitigation by rhizobacteria consortium in wheat field trials. Rhizosphere.

[68] Abbasi M., Sharif S., Kazmi M., Sultan T., Aslam M. (2011). Isolation of plant growth promoting rhizobacteria from wheat rhizosphere and their effect on improving growth, yield and nutrient uptake of plants. Plant Biosyst..

[69] Zafar-ul-Hye M., Danish S., Abbas M., Ahmad M., Munir T.M. (2019). ACC deaminase producing PGPR Bacillus amyloliquefaciens and Agrobacterium fabrum along with biochar improve wheat productivity under drought stress. Agronomy.

